# REST, Exploring Sleep Patterns and Influencing Factors in Elite Female Football Athletes

**DOI:** 10.1038/s41597-025-06331-8

**Published:** 2026-04-09

**Authors:** Matthias Boeker, Andreas Alexandersen, Vajira Thambawita, Cise Midoglu, Dag Johansen, Michael A. Riegler, Pål Halvorsen

**Affiliations:** 1 Simulamet, Oslo, Norway; 2 Oslo Metropolitan University, Oslo, Norway; 3 UiT The Arctic University of Norway, Tromsø, Norway

**Keywords:** Biomarkers, Physiology

## Abstract

This paper presents a novel dataset of 21 elite female football athletes comprising 17 days of actigraphy, well-being, caffeine consumption, screen time, and a daily hand strength test. This dataset aims to provide a comprehensive understanding of the interplay between lifestyle factors, sleep, and athletic performance. Sleep is a crucial process for physical and mental recovery, memory retention, and brain development. The quality of athletes’ sleep is significantly impacted by factors such as rigorous training schedules, stress, light exposure, and caffeine consumption. By examining these factors in detail, this dataset can aid in the development of personalised training models that take into account each athlete’s individual sleep patterns and recovery phases. Such an approach aims to optimise training and recovery strategies to improve athletes’ overall performance and well-being.

## Background & Summary

Professional athletes relentlessly push their bodies and minds each day to improve both their physical and mental abilities, with the extent of their gains mainly restricted by the regeneration time of the human body. The central nervous system’s primary regeneration process is sleep, crucial for recovery and ensuring normal functioning^1^. Previous research underscores the pivotal role of sleep in physical and mental regeneration, injury prevention, energy maintenance, brain development, and immune system function^2^. Studies suggest that sleep aids in consolidating and improving memory of motor skills, which can lead to improved performance after sleep^3^. It is therefore recommended that professional athletes who engage in rigorous training regimes prioritise adequate rest to counterbalance the heightened stress on their bodies. Although sleep is a central process in our lives, there is no single definition of what constitutes optimal and healthy sleep^4^.

Nevertheless, professional athletes and supporting staff are aware of the importance of sleep, yet athletes often experience sleep deprivation due to demanding training schedules, travel commitments, and competitive engagements^5^. In fact, research indicates that intense exercise in the evening can lead to earlier onset of sleep^6,7^. Furthermore, the heightened stress levels experienced by athletes can adversely affect sleep quality, increasing the risk of injury. Research demonstrates that football athletes experienced significantly reduced total sleep time and sleep efficiency during competitive periods compared to training phases^8^. Similarly, another study discovered a negative association between sleep quality and mood, fatigue, and tension at night prior to competition^9^.

The process of sleep is a complex construct and comprises of two primary processes; the sleep-promoting process S (sleep homeostasis), and process C, regulated by the body’s internal clock, known as the circadian rhythm^10,11^ Before the onset of sleep, the process S reaches its peak, which then decreases again during sleep. During wakefulness, sleep pressure accumulates as our brain expends energy through prolonged neuronal activity, leading to adenosine triphosphate depletion and the subsequent buildup of adenosine in the extracellular space. As adenosine accumulates, it binds to receptors in the brain, signaling the increasing need for sleep and recovery (process S). During sleep, adenosine levels decrease, relieving this homeostatic sleep pressure, while process C continues to regulate the timing of sleep and wakefulness according to circadian rhythms^12^.

The circadian rhythm, which regulates the wake-sleep cycle, is influenced by various ‘zeitgebers’ (time givers). These time givers include social cues, light exposure and behaviour^11,13,14^.

Arguably the most important sleep-wake cycle regulator is the hormone melatonin. Melatonin’s principal role is to initiate sleep, primarily influenced by exposure to short-wavelength light on the retina, often referred to as blue light. Blue light exposure, inhibits melatonin production, promoting wakefulness^13^. Consequently, exposure to light during the last part of one’s circadian phase can postpone sleep onset^15^. Research indicates that blue light emitted by digital devices, such as smartphones and screens, which replicates the brightness of the daytime sky, can disrupt the circadian rhythm, thereby impacting sleep patterns^16,17^.

In addition to blue light exposure, one of the most widely used psychotropic drugs to enhance wakefulness is caffeine. Globally, caffeine is the most consumed psychoactive substance and a prevalent stimulant in sports^18^. Caffeine induces wakefulness by delaying the brain’s natural signals of sleep pressure^12^. Clinical studies confirm that caffeine disrupts sleep, affecting both the onset and duration of sleep^19^. Therefore, in today’s digital age of professional sports, where young athletes are increasingly exposed to light-emitting technology and have access to abundant caffeinated beverages, it is crucial to understand athletes’ behaviours concerning these potentially sleep-disrupting factors.

Despite substantial investments in improving athletes’ physical capabilities, there remains a significant gap in understanding and prioritising proper sleep routines and associated lifestyle factors, especially among young female athletes. The primary objective of our data collection was to investigate the sleep behaviour of elite female football athletes to understand the impact of various lifestyle factors on sleep patterns. More specifically, how activity levels, caffeine consumption, and exposure to blue light can influence sleep duration and quality. This dataset aims to support the development of personalised training models that consider individual sleep patterns and recovery phases, thus optimising training and recovery strategies tailored to each athlete’s requirements. Such an approach shows promise for enhancing overall performance and well-being among athletes.

In summary, the main contributions are as follows.

- Providing a detailed examination of sleep habits among elite female football athletes.
- Evaluating the influence of activity levels, caffeine consumption, and blue light exposure on sleep duration and quality.
- Facilitating the creation of individualized training and recovery strategies based on specific sleep patterns and recovery needs.
- Offering insights that can improve overall athletic performance and well-being through optimized sleep and recovery practices.

## Methods

The section describes the procedures and techniques used in the study to collect and analyse data from the participants. The following subsections address the experimental protocol, data collection systems and computational approaches used to preprocess and annotate the data collected during the study. All data collection was approved by the Norwegian Agency for Shared Services in Education and Research (SIKT) under the reference number 604876. All participants provided written informed consent prior to participation, including consent to the open publication of anonymised data in a public repository. The English translation of the informed consent form signed by the participants is attached at the end of the paper as supplementary material (Fig. 6). Given the relatively small sample size, indirect identification of participants cannot be entirely ruled out. This represents a limitation of the dataset, although all identifiers have been removed and the data have been anonymised to the greatest extent possible. This is described in Section Anonymisation.

**Table 1:** Summary statistics of the initial questionnaire focusing on demographic and hand dominance data.

Statistic	Count	Mean	Std Dev	Min	25%	Median	75%	Max
Age	22.0	23.91	4.64	17.0	19.75	23.5	26.75	34.0
Height (cm)	22.0	168.68	5.37	157.0	164.75	169.5	172.0	177.0
Weight (kg)	22.0	66.32	5.30	53.0	63.0	68.0	69.75	75.0
Dominant Hand	22.0	0.32	0.48					
Right Hand	22.0	0.27	0.46					

The table provides a comprehensive overview of counts, central tendency, and variability measures for observed attributes. This summary excludes details from the ASBQ, which should be analysed separately to focus on specific sleep behaviors and their implications on athletic performance.

**Table 2:** Summary statistics of metrics collected in the daily reporting.

Statistic	Count	Mean	Std Dev	Min	25%	50%	75%	Max
Total Sleep Time (min.)	332	474.53	137.44	5.0	439.5	495.0	554.75	773.0
Sleep Intervals	370	3.78	2.61	0.0	2.0	4.0	5.0	13.0
Longest Interval Duration (min.)	332	327.62	150.57	5.0	222.5	315.0	453.0	773.0
Sleep Period Mean	332	17.47	5.67	3.49	15.26	17.35	20.46	43.83
Sleep Quality	273	3.40	0.98	1.0	3.0	3.0	4.0	5.0
Fatigue	278	2.71	0.56	1.0	2.0	3.0	3.0	4.0
Soreness	273	2.35	0.60	1.0	2.0	2.0	3.0	4.0
Readiness	280	6.43	1.67	1.0	6.0	7.0	8.0	10.0
Self-Reported Sleep Duration (h)	280	7.94	1.37	2.0	7.0	8.0	9.0	12.0
Caffeine Intake (mg)	370	90.28	104.83	0.0	0.0	65.0	160.0	765.0

This table provides an overview of counts, central tendency, and variability measures of sleep patterns, quality, and associated behavioral factors such as caffeine intake.

### Experimental protocol

Twenty-one participants (23.91 ± 4.64; range 17–34 years old) volunteered in the experiment. All participants are members of the same team of a Norwegian football club. The experiment was conducted during spring 2023. The participants recorded activity by a wrist-worn ActiGraph™ wGT3X-BT sensor. Additionally, the experiment included two questionnaires, an initial and a daily one. The initial questionnaire was conducted before the sensor recording started. The daily questionnaire was filled out every morning for the previous day, thus covering the perceived sleep quality of the night and daily routines of the previous day. The team uses a performance tracking tool called XPS™ in which participants report matchdays, wellness parameters such as *readiness*, *soreness* and *fatigue* (https://www.sidelinesports.com/no/). Participants could indicate *soreness* on up to two body locations of perceived soreness, which are recorded in the dataset as soreness_location_1 and soreness_location_2. Matchdays are coded using the MD-X periodization system. MD (match day), MD-1/-2 1-2 days before match, MD+1/+2 (recovery phase, 1-2 days after match).

**Fig. 1:**
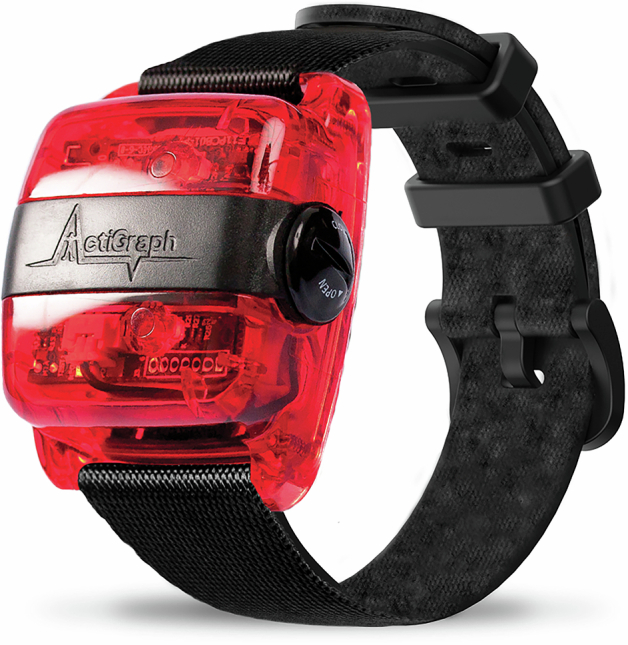
An example image of the ActiGraph™ wGT3X-BT sensor, which was used for experiment. The image is taken from the official Actigraph webpage https://theactigraph.com/actigraph-wgt3x-bt.
- **Initial questionnaire:** The one-time initial questionnaire contains demographic questions and the Athlete Sleep Behaviour Questionnaire (ASBQ). The questionnaire contextualises the individual sleep patterns and provides a comprehensive understanding of them. The ASBQ was developed and validated by Driller *et al*.^20^ to understand sleep behaviour and habits. The questionnaire is specially tailored for athletes and their most common sleep issues. Responses to each question are measured using a Likert scale, where 1 signifies ‘*‘never’*, 2 denotes *‘rarely’*, 3 represents *‘sometimes’*, 4 corresponds to *‘frequently’*, and 5 indicates *‘always’*. The 18 item questionnaire revealed three latent factors; routine/ environmental factors, behavioural factors, and sport-related factors^20^. The initial questionnaire is presented in Table 3.

Furthermore, the participants measured their handgrip strength daily with a handheld dynamometer, which is reported in kilograms. Measurements were conducted each morning using the dominant hand consistently throughout the study period, supervised by the team physiotherapist. Detailed equipment specifications and standardized measurement protocols were not fully documented, as measurements were conducted by team staff following standard practice. In short, we have the following data sources:

- **Sensor data:** An ActiGraph™ wGT3X-BT sensor was attached to the most convenient wrist of each participant. Figure 1 shows a picture of the sensor from the official ActiGraph website (https://theactigraph.com/actigraph-wgt3x-bt). The sensor recordings started and ended simultaneously. It was critical to ensure that the sensor should not disturb the normal behaviour and sleep pattern of the participant, and that the location of the sensor remained unchanged throughout the duration of the experiment, in order to maintain consistency in data collection. The sensor was taken off during showers, bathing, or any activity with extensive water contact. For the athletes safety, the sensors were also removed during football training and matches. The sensor recorded acceleration in three dimensions at 100*Hz* to register movement. The data was stored directly on the devices’ internal storage. The battery-life of the sensors lasts over 20 days. This makes the sensors suitable for long time observations of activity and sleep patterns.

**Table 3:** Initial questionnaire.

No	Question	Mandatory	Answer Options
1	Id	*✓*	Text field (numeric)
2	Age	*✓*	Text field (numeric)
3	Your height in centimeters (cm)	*✓*	Text field (numeric)
4	Your weight in kilograms (kg)	*✓*	Text field (numeric)
5	Placement of the sensor (left or right, and if dominant hand)	*✓*	Left hand (dominant hand) /
			Left hand (NOT dominant hand) /
			Right hand (dominant hand) /
			Right hand (NOT dominant hand)
6	Do you use Nicotine regularly (Ex: smoking, snus)?	*✓*	Yes / No
7	Do you use contraceptives (containing estrogen, progesterone)?	✗	Yes / No / Prefer not to answer
8	Athlete Sleep Behaviour Questionnaire (ASBQ)		
8.1	I take afternoon naps lasting two or more hours		
8.2	I use stimulants when I train/compete (e.g caffeine)		
8.3	I exercise (train or compete) late at night (after 7pm)		
8.4	I consume alcohol within 4 hours of going to bed		
8.5	I go to bed at different times each night (more than ± 1 hour variation)		
8.6	I go to bed feeling thirsty		
8.7	I go to bed with sore muscles		
8.8	I use light-emitting technology in the hour leading up to bedtime (e.g laptop, phone, television, video games)	*✓*	Never / Rarely / Sometimes / Frequently / Always
8.9	I think, plan and worry about my sporting performance when I am in bed		
8.10	I think, plan and worry about issues not related to my sport when I am in bed		
8.11	I use sleeping pills/tablets to help me sleep		
8.12	I wake to go to the bathroom more than once per night		
8.13	I wake myself and/or my bed partner with my snoring		
8.14	I wake myself and/or my bed partner with my muscle twitching		
8.15	I get up at different times each morning (more than ± 1 hour variation)		
8.16	At home, I sleep in a less than ideal environment (e.g too light, too noisy, uncomfortable bed/pillow, too hot/cold)		
8.17	I sleep in foreign environments (e.g hotel rooms)		
8.18	Travel gets in the way of building a consistent sleep-wake routine		
9	Do you use any sleep medication? (Melatonin, others)	✗	Yes / No / Prefer not to answer
10	Comments (optional)	✗	Text field

Questions 7.1-7.18 form the Athlete Sleep Behaviour Questionnaire (ASBQ).
- **Daily reporting:** Daily reporting included the daily questionnaire designed for the experiment and the XPS reporting system. The daily questionnaire contained questions about screen time, the timing and the amount of caffeine intake, and sleep quality. The aim was to understand of how screen time and caffeine intake affected sleep quantity and quality. The participants reported their caffeine consumption in terms of identifiable units such as energy drinks, cups of coffee, or shots of espresso. A detailed calculation of caffeine in *mg* per day is rather difficult and cumbersome, it might lead to inconsistent results among the participants due to calculation errors or inaccurate reporting. Thus, we used a simplified questionnaire to focus on the number of units consumed. In addition, we collected information about the time of last caffeine consumption in order to gain a more comprehensive understanding of consumption patterns. The daily questionnaire is presented in Table 4.

**Table 4:** Daily questionnaire.

No	Question	Mandatory	Answer Options
1	How much time did you spend in front of a screen in the hour before you fell asleep? (Mobile, PC, TV)		No time /
			Less than 15 minutes /
		*✓*	Around 30 minutes /
			45 minutes or more /
			I don’t remember
2	Did you drink any caffeine today? If not, leave blank		
2.1	Energy drinks (0,5l)		
2.2	Cola drinks (0,5l)	✗	1 / 2 / 3 / 4 or more
2.3	Cup of Coffee (ca 0,2l)		
2.4	Cup of Tea (0,2l)		
2.5	Espresso shot (Latte, Cappuchino)		
3	At what time did you consume most of the caffeine?		Morning (before 12:00 pm) /
		✗	Afternoon (between 12:00 - 18:00) /
			Evening (after 18:00)
4	How well did you sleep last night? (Sleep quality, not length)		1 (Not good) /
			2 /
		*✓*	3 /
			4 /
			5 (Very good)
5	How fatigued do you feel?		1 (Very fatigued) /
			2 /
		*✓*	3 /
			4 /
			5 (Not fatigued)
6	How sore are your muscles?		1 (Very sore) /
			2 /
		*✓*	3 /
			4 /
			5 (Not sore at all)
7	How ready are you to train?		1 (Not ready to train) /
			2 /
		*✓*	…/
			9 /
			10 (Very ready to train)
8	How long did you sleep?	*✓*	hours

All data collection has been approved by the Norwegian Agency for Shared Services in Education and Research (SIKT) under the reference number 604876. The participants gave their informed consent. The English translation of the informed consent form signed by the participants before the study was conducted is attached at the end of the paper.

### Computational preprocessing

The raw acceleration signal has been filtered to reduce noise and artifacts. According to Actigraph, the official bandpass filtering frequencies are 0.25*Hz* and 2.5*Hz*. However, the bandpass order number has not been published. We applied a third-order bandpass filter, following results from Van Hess *et al*.^21^, who investigated the bandpass filter performances for Actigraph sensors. Activity data in sleep studies are usually provided in epochs. An epoch accumulates the activity for a certain time window. The epoch length is 30 seconds. The acceleration signal per epoch is rectified and summed up to the so called activity counts. These epochs are derived for each acceleration dimension and the vector magnitude in Equation (1):

1 $$VM=| V| =\sqrt{{x}^{2}+{y}^{2}+{z}^{2}}$$

### Anonymisation

To ensure the anonymity of the participants in this study, names were not digitally recorded at any point during data collection or analysis. In addition, all dates were removed from the dataset to prevent possible identification of individuals. The exact dates were replaced by weekday indicators and the 24h-notation timestamps were kept to retain the time dimension (representativeness with respect to diurnal and day-of-week patterns). These measures were taken to protect the privacy of the participants.

### Annotation

According to sleep research, the most reliable method to determine sleep is Polysomnography (PSG). In PSG, the participants’ neurological signals are recorded, which are then analysed and annotated by experts in relation to sleep^22^. However, PSG is an invasive, expensive, and cumbersome technique. Since actigraphy is an objective and robust method to record human activity, it has been proposed to also be able to detect sleep patterns based on movements during sleep. Actigraphy is not as accurate as PSG, but it is non-invasive, cost effective, and can be applied over several weeks to observe long term sleep patterns. To detect sleep through actigraphy, a variety of sleep detection algorithms has been developed. For this dataset, we provide sleep annotation from the Sadeh, Cole-Kripke, Oakley sleep algorithm, and transfer learning approaches. The ActiGraph™ software offers the algorithms of Sadeh and Cole-Kripke. Cole-Kripke *et al*. used regression analysis for the classification of sleep^23^. Sadeh *et al*. performed discriminant analysis to classify sleep^24^. Oakley’s method applies a threshold to distinguish between sleep and wakefulness. The transfer learning approach trains a Long Short Term Memory (LSTM) on sleep data of the Multi-Ethnic Study of Atherosclerosis (MESA). The MESA contains actigraphy with annotated sleep based on PSG. The PSG is used to label supervised learning. The LSTM was trained on 250 participants. For each participant, we split the actigraphy in train (90%) and test (10%) sets. With the trained LSTM, we infer sleep for the here presented data to generate pseudo-labels. Table 5 compares the classification performance of traditional algorithms with LSTM. The classification performance is derived from the test set of the MESA study. Sleep states in the resulting data are coded as: s = sleep, w = wake, n = non-wear.

**Table 5:** The performance metrics of different sleep detection algorithms (LSTM, Oakley, Sadeh, and Cole-Kripke) as evaluated in the MESA.

Metric	LSTM	Oakley	Sadeh	Cole-Kripke
Sensitivity	0.873	0.931	0.937	0.813
Specificity	0.883	0.956	0.837	0.987
MCC	0.740	0.889	0.748	0.825
Kappa	0.734	0.888	0.726	0.814
F1	0.845	0.936	0.842	0.886
Accuracy	0.878	0.944	0.887	0.900

These metrics include Sensitivity, Specificity, Matthews Correlation Coefficient (MCC), Kappa, F1 score, and Accuracy. Each column represents a different algorithm, and each row displays the values of these metrics, allowing for a comparative analysis of their effectiveness in detecting sleep.

In addition to sleep and wake annotation, we provide annotation of non-wear time (time where sensor was not applied to the wrist). As mentioned in Section, the sensor was not applied during football training, match days, or activities with extensive water exposure. A threshold based algorithm detects non-wear times. The threshold value was set based on the sensor’s base signal when not applied.

## Data Records

**Fig. 2:**
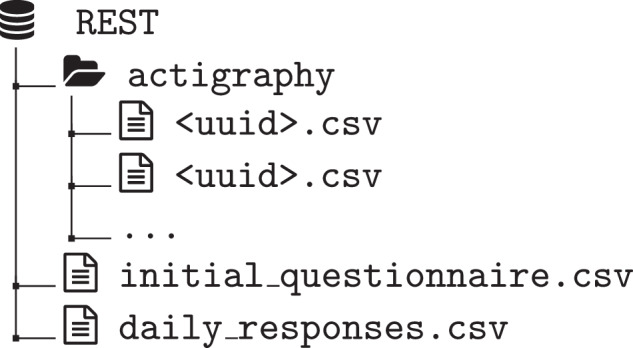
The figure depicts the folder structure and file naming of the dataset.

The REST dataset is available from Zenodo^25^. The license for the dataset is Creative Commons Attribution 4.0 International (CC BY 4.0). It contains sleep analysis data that was collected from 21 participants over a period of 17 days, during which the participants’ activity was monitored, and their responses to daily questionnaires were systematically recorded. The three different data types collected are; (i) responses from the initial questionnaire, (ii) responses from the the daily questionnaires, and (iii) the recorded actigraphy sensor data. All files are provided as comma-separated values (csv) files. While the csv file with the collected answers of the initial questionnaire and the daily answers contains all participants, the actigraphy is saved as a csv file for each participant individually. Figure 2 visualises the folder structure of the dataset.

- **Sensor data:** The sensor-based actigraphy data is stored in the actigraphy folder and each file is named after the participant ID. Table 6 shows the column description for the actigraphy files. The files contain 30-second epoch activities in the three dimensions *x*, *y*, *z*. In addition, the file contains sleep annotations from 4 different algorithms. Each of the 21 actigraphy files within the dataset contains nine variables. Each file contains a total of 48,480 records that provide a detailed overview of physical activity patterns.

**Table 6:** The table describes the variables for the daily-responses.csv file and the actigraphy files.

File Name	Column Name	Description
daily-responses.csv	id	Unique identifier for each entry.
	weekday	Day of the week.
	longest_interval_duration	The longest period of sleep (sensor).
	sleepdura_sr	Self reported sleep duration (hours).
	sleepquality	Sleep quality from 1-5.
	total_sleep_time	Total sleep time (sensor).
	longest_interval_start	Start time of the longest sleep interval.
	longest_interval_end	End time of the longest sleep interval.
	sleep_intervals	Number of sleep intervals (sensor).
	sleep period mean	Mean activity during longest sleep period (sensor).
	caffeine_mg	Sum of caffeine (mg) intake.
	caffeine_time	Last time point of caffeine intake.
	screentime	Screen time an hour before sleep.
		(short: <15min, medium: 15-45min, long: >45min).
	sleep_onset	Sleep onset (sensor).
	sleep_offset	Sleep offset (sensor).
	sleep period standard deviation	Standard deviation of activity during longest sleep period (sensor).
	sleep period msd	Mean squared deviation of activity during longest sleep period (sensor).
	Comments (optional)	Comments (Norwegian).
	energy	Units of energy drinks (0.5l).
	cola	Units of cola drinks (0.5l).
	coffee	Coffee cups (0.2l).
	tea	Tea cups (0.2l).
	espresso	Units of espresso (0.04l)
	handgrip_kg	Handgrip strength (kg).
	matchday	Is the day a matchday.
	fatigue	How fatigue do you feel from 1-5.
	soreness	How sore are your muscles from 1-5.
	soreness_location_1	Location of soreness
	soreness_location_2	Location of soreness
	readiness	How ready are you to train from 1-10.
Actigraphy file	x, y, z, vm	Activity in the three dimensions and vector magnitude.
	time	Time in 30 second epochs.
	weekday	Day of the week.
	CK	Cole-Kripke algorithm predictions.
	Oakley	Oakley algorithm predictions.
	Sadeh	Sadeh algorithm predictions.
	fourier_HMM	Hidden Markov model predictions.
	LSTM	LSTM algorithm predictions.
- **Initial questionnaire:** The responses to the initial questionnaire are gathered for all participants in the file
  initial-questionnaire-responses.csv. Table 1 contains summary statistics of the demographic variables of the initial questionnaire. The majority with 73% chose the left hand to wear the sensor, with 32% of participants wearing the sensor on their dominant hand.
- **Daily reporting:** The daily responses are stored in daily-responses.csv for each day and participant. Additionally to the subjective daily responses of the participant, the file contains objective sleep metrics obtained from the actigraphy data. Overall, the data contained in the file includes a comprehensive range of 29 variables with in total 273 responses that provide a comprehensive overview of the participants’ daily experiences and responses. Table 6 shows the variables and descriptions of the daily-responses.csv. Sleep metrics derived from actigraphy are marked with (*sensor*) in Table 6. Table 2 describes summary statistics of the data from the daily reporting.

## Technical Validation

In the first part of the technical validation, we explore and visualise the objective actigraphy data. In the second part, we demonstrate the relationship between subjective and objective sleep metrics for validation.

### Exploration of actigraphy data

**Fig. 3:**
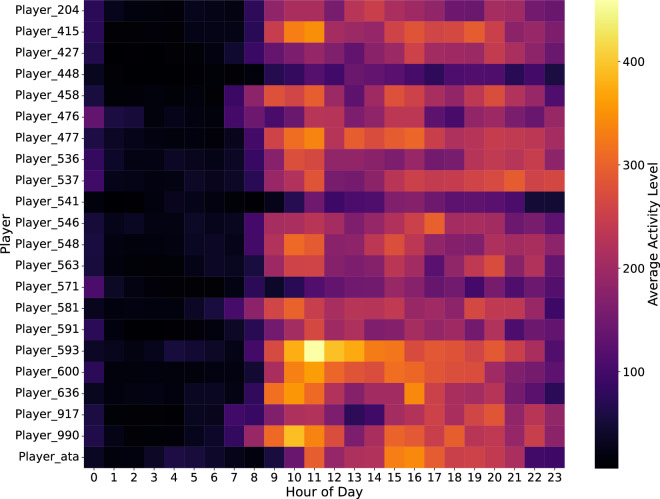
The average hourly activity levels of various athletes and averaged across 17 days. The athletes are listed along the vertical axis and the hours of the day are represented on the horizontal axis, ranging from 0 to 23. The colour intensity within the heatmap corresponds to the average activity level of each player at each hour of the day. The plot highlights variations in activity patterns among athletes, showing periods of higher and lower activity throughout the day.

Visual analysis of the actigraphy reveals daily patterns and gives insights into the circadian rhythm of the athletes. Figure 3 visualises the averaged hourly activity of the athletes. In the heatmap, warmer colours represent higher average activity levels during each hour. As shown in Fig. 3, the activity patterns of the athletes are different. Some athletes have a consistently higher level of activity throughout the day, while others have clear phases of high and low activity. The differences between athletes in terms of when periods of low activity occur indicate sleep schedules. Most of the athletes begin to be active around 8 AM. Irregular sleep patterns potentially impact restfulness and recovery. Sporadic instances of higher activity during typical sleep periods indicate interruptions in sleep. These could result from awakenings and possibly affect sleep quality.

**Fig. 4:**
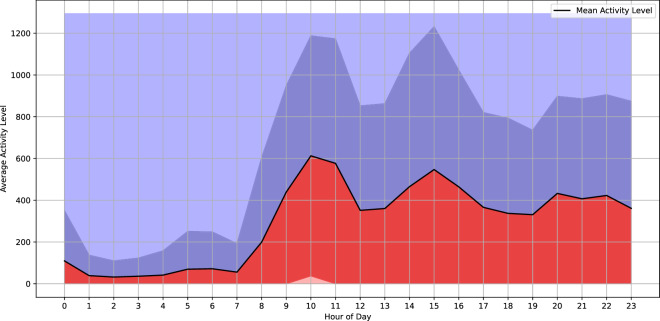
The 24-hour activity profile of player 593, illustrating both the averaged activity levels and their standard deviation throughout the day. The mean activity level is depicted by a solid black line, with shaded red and blue areas representing the standard deviation around the mean, thereby indicating the variability in activity levels at different hours. This visualisation provides insight into the player’s daily activity patterns, including periods of higher and lower activity, as well as the consistency of these patterns across the day.

Figure 4 shows a more detailed analysis of the daily sleep pattern. The figure portraits the averaged 24 hour activity profile with standard deviation of player 593. The graph shows the mean activity level as a black line for each hour of the day, with the dark blue shaded area above indicating upper standard deviation and the dark red shaded area below the lower standard deviation. The 24h profile helps us to better understand an individual’s activity patterns. Figure 4 shows that the player is usually active from 7 AM and reaches its peak at lunchtime. Activity decreases in the afternoon.

### Relationship between subjective and objective sleep metrics

**Fig. 5:**
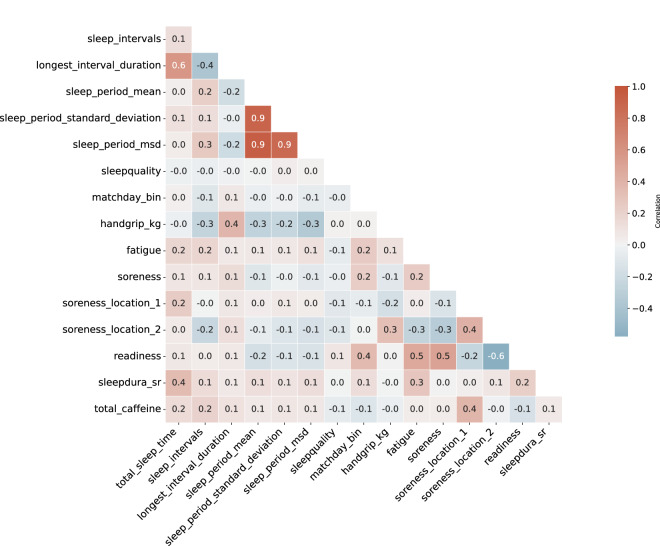
Spearman’s rank correlation matrix of the ordinal and numeric variables of the daily reports. The matrix combines subjective and objective variables, showing the correlation coefficients between various aspects of sleep quality, physical performance indicators, and other factors such as caffeine intake. The colours range from red (positive correlation) to blue (negative correlation), with the scale indicating the strength of the correlation.

The daily reports contain a combination of subjective and objective variables. The variables are of different nature, ordinal, numeric, and categorical. Spearman’s rank correlation analysis allows for analysis of correlation between numeric and ordinal variables, shown in Fig. 5. Interestingly, isometric handgrip strength appears to be correlated to objective sleep metrics. We observed a positive correlation with longest sleep interval (typically indicative of stable sleep), and a negative correlation with a high number of sleep intervals (typically indicative of disrupted sleep). Match day is a binary indicator if the day was an official match day (not training). Match days are especially correlated with the readiness of a player. Total sleep time is correlated with subjective self-reported sleep.

**Fig. 6:**
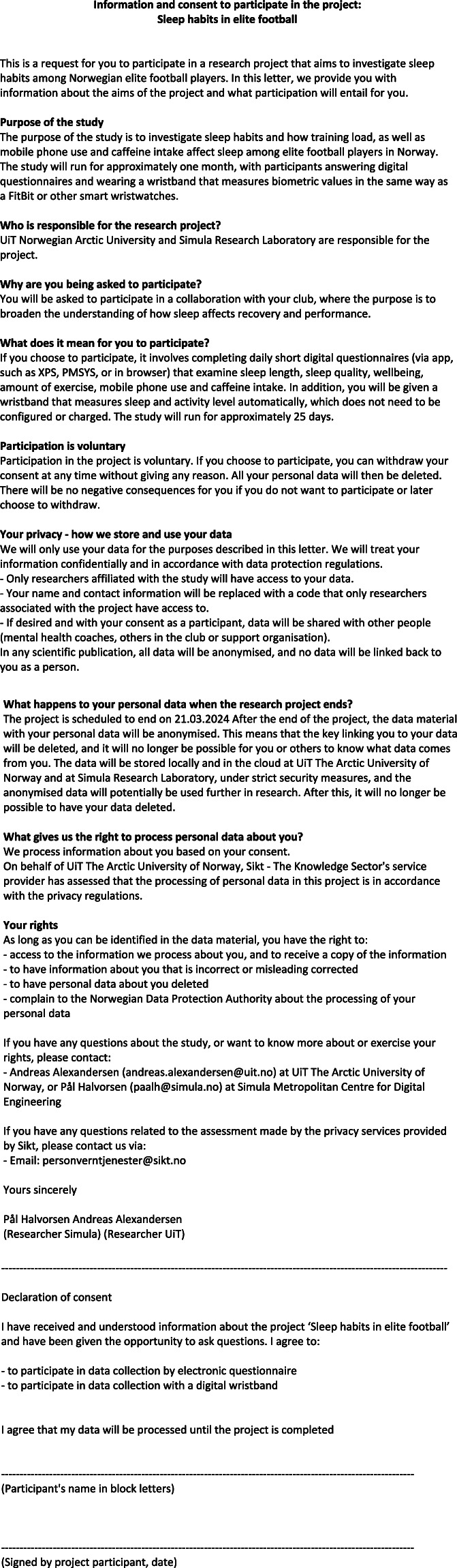
English version of the informed consent form signed by the participants before the study was conducted.

After exploring correlations among subjective and objective variables, we demonstrate statistically significant relationships between subjective and objective measures of sleep time. We examine the fixed effects of subjective sleep duration and sleep quality as well as the quadratic effect of sleep quality on the dependent variable: objectively measured total sleep time. In this model, participants are treated as a random effect to account for between-subject variability. Equation (2) shows linear mixed effects model. Table 7 shows the results of the linear mixed effects models. The subjective sleep duration has a significant fixed effect on objective total sleep. While subjective sleep duration is represented as an integer in hours, total sleep time is given in minutes and divided by 60. Thus we can interpret the coefficient of 0.523, as the increase of one hour of subjective sleep quality changes the total sleep time by half an hour. Self-reported subjective sleep was reported as whole hours, thus rounding to the nearest hour was expected. Furthermore, while there does not appear to be a significant linear relationship between sleep quality and total sleep time, the quadratic effect of sleep quality is significant, indicating a non-linear relationship:

2 $$\,{\rm{total\; sleep\; time}}={\beta }_{0}+{\beta }_{1}{\rm{sleep\; duration}}+{\beta }_{2}{\rm{sleep\; quality}}+{\beta }_{3}{{\rm{sleep\; quality}}}^{2}+{u}_{j}{\rm{participant\; id}}$$

**Table 7:** Mixed linear model regression results.

Parameter	Coef.	Std.Err.	z	P > |z|	95% CI
Intercept	259.571	48.226	5.382	0.000	[165.050 354.092]
Sleep duration (subj.)	0.520	0.098	5.296	0.000	[0.327 0.712]
Sleep quality (subj.)	−3.945	8.800	−0.448	0.654	[−21.193 13.303]
(Sleep quality)^2^ (subj.)	−14.374	6.323	−2.273	0.023	[−26.767 −1.981]
Group Var	765.409	7.233			

## Limitations

The REST dataset has several possible limitations. Firstly, the data was only collected from one team, and might not be fully representative of all female football athletes. Secondly, the data was collected for a short period of time and does not cover a full yearly cycle. Thus, it does not include time periods with different levels of natural light (e.g., periods with more daylight especially in the Scandinavian countries). Thirdly, several players reported that they got disturbed by the LED indicator of the actigraphy device, and taped over the LED in order to minimise the impact of this blinking light on their sleep. Such disturbances might have affected their sleep quality. Four athletes did not report matchday and wellness parameters through the XPS system, resulting in incomplete entries for these variables in the daily responses file. These missing entries represent non-compliance with the team’s reporting system rather than specific training status or injury absence.

## Data Availability

The sleep patterns of female football athlete data is available at Zenodo 10.5281/zenodo.16937033. The license for the data is Creative Commons Attribution 4.0 International (CC BY 4.0).

## Code availability

The code repository with the scripts for data preprocessing, the different annotation algorithms, as well as the technical validations are available at https://github.com/simula/REST.
